# Methodological Rigour and Transparency of Clinical Practice Guidelines Developed by Neurology Professional Societies in Croatia

**DOI:** 10.1371/journal.pone.0069877

**Published:** 2013-07-19

**Authors:** Katarina Ivana Tudor, Petra Nimac Kozina, Ana Marušić

**Affiliations:** 1 Department of Neurology, Zagreb University Hospital Centre, Zagreb, Croatia; 2 Department of Research in Biomedicine and Health, University of Split School of Medicine, Split, Croatia; Universidad Peruana de Ciencias Aplicadas (UPC), Peru

## Abstract

**Background:**

Clinical practice guidelines are systematically created documents that summarize knowledge and assist in delivering high-quality medicine by identifying evidence that supports best clinical care. They are produced not only by international professional groups but also by local professionals to address locally-relevant clinical practice. We evaluated the methodological rigour and transparency of guideline development in neurology formulated by professionals in a local medical community.

**Methods:**

We analyzed clinical guidelines in neurology publicly available at the web-site of the Physicians’ Assembly in Croatia in 2012: 6 guidelines developed by Croatian authors and 1 adapted from the European Federation of Neurological Societies. The quality was assessed by 2 independent evaluators using the AGREE II instrument. We also conducted a search of the Cochrane Library to identify potential changes in recommendation from Cochrane systematic reviews included in guideline preparation.

**Results:**

The methodological quality of the guidelines greatly varied across different domains. „Scope and Purpose” and „Clarity of Presentation“ domains received high scores (100% [95% confidence interval (CI) 98.5–100] and 97% [77.9–100], respectively), the lowest scores were in “Stakeholder Involvement“ (19% [15.5–34.6]) and “Editorial Independence” (0% [0–19.2]). Conclusions of 3 guidelines based on Cochrane systematic reviews were confirmed in updated versions and one update provided new information on the effectiveness of another antidepressant. Two Cochrane reviews used in guidelines were withdrawn and split into new reviews and their findings are now considered to be out of date.

**Conclusion:**

Neurological guidelines used in Croatia differ in structure and their methodological quality. We recommend to national societies and professional groups to develop a more systematic and rigorous approach to the development of the guidelines, timely inclusion of best evidences and an effort to involve target users and patients in the guideline development procedures.

## Introduction

Clinical practice guidelines (CPGs) are systematically created documents that summarize the knowledge and assist in delivering high-quality medicine by identifying evidence that supports the best clinical care [1]. They are produced not only by international organizations but also by professional associations in many countries, aiming to supply the local professionals with recommendations based on currently available evidence for best standard of care for the patients [2]–[4].

In order to ensure that guidelines are valid and relevant for practice, it is important that they are prepared in a methodologically appropriate process. For this purpose, procedures to assess the methodological quality of CPGs have been developed, most notably the Appraisal of Guidelines for Research and Evaluation (AGREE) instrument [5]. AGREE instrument is a widely recognized tool for guideline evaluation of various fields of medicine [6]–[8].

Neurology is a rapidly developing field where the new and relevant evidence may have important implications for patients’ health care and outcome prognosis. However, there are only a few guidelines for practice in neurology which been evaluated using a standardized instrument [2], [9]. Our aim was to evaluate the methodological rigour and transparency of guideline development in neurology as formulated by professionals in a local medical community. We used the advantage of public access to clinical guidelines in neurology developed by the neurology experts from the Physicians’ Assembly in Croatia, which has a tradition in developing and maintaining professional standards in Croatia since 1874 [10]. We also evaluated how many guidelines were based on Cochrane systematic reviews, considered to be the evidence of highest quality [11], [12], and whether there would be a change in recommendations based on recent evidence.

## Methods

We analyzed 7 neurology guidelines that were available at the web-site of the Croatian Physicians’ Assembly in 2012 [10]. Six were developed by Croatian authors and one was adapted (translated) from the European Federation of Neurological Societies (EFNS) guideline: 1) Consensus opinion on diagnosing brain death – Guidelines for use of confirmatory tests [13]; 2) Recommendations for neuropathic pain treatment [14]; 3) Recommendations for stroke management 2006 update [15]; 4) Evidence based guidelines for treatment of primary headaches [16]; 5) Guidelines for preoperative diagnostic evaluation of patients with pharmacoresistant epilepsy [17], 6) Epilepsy – therapeutic guidelines [18], 7) EFNS guidelines on pharmacological treatment of neuropathic pain [19].

We used The Appraisal of Guidelines for Research and Evaluation II (AGREE II) instrument [20] to assess the quality of guidelines. The instrument has 23 items, grouped in 6 quality domains: 1) Scope and Purpose (items 1–3), 2) Stakeholder Involvement (items 4–6), 3) Rigour of Development (items 7–14), 4) Clarity of Presentation (items 15–17), 5) Applicability (items 18–21) and 6) Editorial Independence (items 22–23). Each item was rated on a 7-point scale: from 1– strongly disagree to 7– strongly agree.

Two assessors independently performed the rating of guidelines. Each of two evaluators also independently judged the overall quality of the guideline from 1 (least quality) to 7 (highest quality). They independently submitted their scores to the third investigator, who then calculated the overall scores [5]. The overall score for each domain was calculated by summing up all the scores of the individual items in the domain and the total was standardised as a percentage of the maximum possible score for that domain. The following formula was used:

The scaled domain score = (Obtained score − Minimum possible score)/(Maximum score − Minimum possible score)×100.

where “Obtained score” was the sum of the scores by individual assessors, Maximum score  = 7 (strongly agree)×2×No. items in the domain, and minimum score  = 1 (strongly disagree)×2×No. items in the domain [5].

The formula was also used to calculate the overall quality score.

We searched the Cochrane Database of Systematic Reviews to identify any potential change of recommendation from the version of the Cochrane systematic reviews included in guideline preparation. The search strategy included the terms from respective guidelines. The date of the Cochrane Library search was 13 May 2012.

## Results

Out of 7 guidelines, 2 were published within 2 years before the time of analysis, and 4 were published in 2005 or 2006 (**Table 1**). Only a single guideline stated that it was an update of a previous version, and 3 explicitly mentioned the method of development (**Table 1**).

**Table 1:** Characteristics of Croatian neurology guidelines.

Guideline	Year of publication	Update/period	Development method	Number of references	Topics covered
Consensus opinion on diagnosing brain death – Guidelines foruse of confirmatory tests	2005	Not mentioned	Not mentioned	58	Diagnosis
Recommendations for neuropathic pain treatment	2008	Not mentioned	Not mentioned	122	Treatment
Recommendations for stroke management, 2006 update	2006	First published in 2001	Literature review and consensus	507	Diagnosis/Treatment
Evidence based guidelines for treatment of primary headaches	2005	Not mentioned	Literature review and consensus	235	Diagnosis/Treatment
Guidelines for preoperative diagnostic evaluation of patients with pharmacoresistant epilepsy	2010	Not mentioned	Not mentioned	30	Diagnosis
Epilepsy – therapeutic guidelines	2010	Not mentioned	Not mentioned	34	Treatment
EFNS guidelines on pharmacological treatment of neuropathic pain*	2006	2 year update period announced in the article	Literature review and consensus	142	Treatment

* Croatian translation of a guideline from EFNS – European Federation of Neurological Societies.

The highest scores for all guidelines were observed in the domains “Scope and purpose” and “Clarity of Presentation”, and there was not much variability in the score among the guidelines (**Table 2**). The lowest scores (zero values) were given to “Editorial Independence” for all locally developed guidelines, except for EFNS guidelines [19] on pharmacological treatment of neuropathic pain (**Table 2**).

**Table 2:** Domain scores for 7 neurology clinical guidelines of the Croatian Physicians’ Assembly.

Guideline*	Domain score (%)†						Overall assessment (%)
	Scope and purpose	Stakeholder involvement	Rigor of development	Clarity and presentation	Applicability	Editorial independence	
Consensus opinion on diagnosing brain death – Guidelines for use of confirmatory tests	97	47	38	100	25	0	78.5
Recommendations for neuropathic pain treatment	100	19	13	89	15	0	78.5
Recommendations for stroke management, 2006 update	100	17	46	100	25	0	85.7
Evidence based guidelines for treatment of primary headaches	100	14	10	97	35	0	78.5
Guidelines for preoperative diagnostic evaluation of patients with pharmacoresistant epilepsy	100	22	25	67	29	0	78.5
Epilepsy – therapeutic guidelines	100	17	32	97	25	0	78.5
EFNS guidelines on pharmacological treatment of neuropathic pain	100	19	94	100	52	38	100
Median (95% confidence interval)	100 (98.5–100)	19 (15.5–34.6)	32 (11.5–70.2)	97 (77.9–100)	25 (19.9–43.6)	0 (0–19.2)	78.5 (78.5–92.9)

EFNS – European Federation of Neurological Societies.

The domain “Stakeholder Involvement” also received low grades because the views and preferences of the target population (patients, public, etc.) were not often sought. The target users of the guideline were clearly defined only in the Consensus opinion on diagnosing brain death – Guidelines for use of confirmatory tests [13].

The scores in the domain “Applicability” were below 50% for all guidelines, because they did not have clear explanation of existing facilitators and barriers that could impact the application of guideline recommendations (**Table 2**). Again, EFNS guidelines on pharmacological treatment of neuropathic pain [19] fared better on this domain and received the composite score of 52% (**Table 2**).

The scores for the domain “Rigour of Development” varied across the guidelines (**Table 1**). Details of the strategy used to search for evidence were described in EFNS guidelines on pharmacological treatment of neuropathic pain [19] and in the Consensus opinion on diagnosing brain death – Guidelines for use of confirmatory tests [13]. The criteria for selecting the evidence was described only in the EFNS guidelines [19]. Methods for formulating recommendations were described in EFNS guidelines on pharmacological treatment of neuropathic pain [19] and in Recommendations for stroke management 2006 update [15]. We found no evidence of external review prior to the publication for any of the evaluated guidelines. A clear statement about the procedure for updating the guideline was provided only in the EFNS guideline [19]. The median score for “Overall Assessment” was 78.5% for Croatian guidelines, whereas the EFNS guideline achieved the full overall assessment score (**Table 2**).

**Figure 1:**
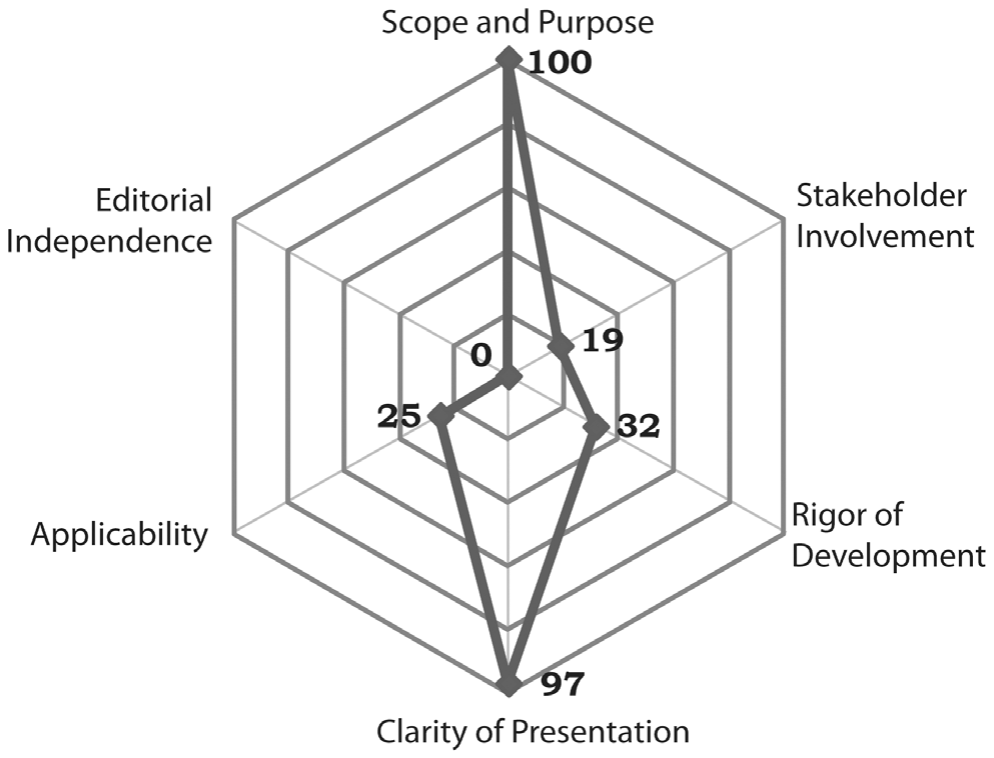
Median scores of evaluated guidelines in five domains of AGREE II instrument.

The median score for of all guidelines was almost maximal for the “Scope and Purpose” and” Clarity and Presentation” domains and below 32% for other domains except Editorial independence which was 0 (**Figure 1**).

The inter-rater agreement (weighted kappa) between the two raters ranged from good (0.725, standard error 0.1449) to very good (0.949, standard error 0.051) across domains in individual guidelines [21].

In 3 out of 7 assessed guidelines, recommendations were partially based on evidence from Cochrane systematic reviews: EFNS guidelines on pharmacological treatment of neuropathic pain [19]; Recommendations for neuropathic pain treatment [14]; and Recommendations for stroke management 2006 update [15]. The key conclusions made by prior studies were confirmed in updated versions of the reviews and one update provided new information on effectiveness of another antidepressant – venlafaxine [22]. Additionally, 2 Cochrane reviews whose results were included in EFNS guideline guidelines on pharmacological treatment of neuropathic pain [19] and Recommendations for neuropathic pain treatment [14] were withdrawn and split into new reviews and their findings are now considered to be out of date [23], [24]. Only a single guideline [19] had a procedure for updating the evidence for the recommendations.

## Discussion

Our evaluation of the methodological rigour and transparency of publicly available neurological guidelines used in Croatia demonstrated a general lack of structured procedure for guideline preparation, as well as significant deficiencies in the following domains: “Applicability”, “Editorial Independence” and “Stakeholder Involvement”. A single guideline provided information about the procedure for its updating [19], and most of the guideline were older than 4 years, which is close to the estimated “half life” of 5.5 years for systematic reviews [25]. Even for more recent guidelines we identified a change in the evidence base [23], [24], i.e. withdrawal of Cochrane systematic reviews because the reviews were not updated.

The limitations of the study include the small number of evaluated guidelines so we did not attempt any statistical conclusion. Our aim was to assess only publicly available guidelines supported by professional groups and societies, as those were considered to be most influential and relevant for the clinical practice in Croatia. Although the AGREE instrument has been tested for validity in different clinical areas and by different professionals [26], its semiquantitive nature of the instrument does not allow conclusions on what is of high or low quality, so that the results of this study have to interpreted with caution and in the context of the local practices and health care system. It is also important to keep in mind that the AGREE instrument only evaluates how the individual items within separate evaluation domains were reported, which may create a bias if the individual items were included during the development of the guideline but were not reported. However, it is likely that guidelines that are better reported contain more appropriate and relevant recommendations. Finally, the instrument does not evaluate the validity of the actual recommendations in the guidelines. The raters were neurology residents without experience in guideline development and they were not blinded to the source and authors of the guidelines. This may have introduced bias, as it was shown that the instrument had low reliability when used by untrained professionals in developing, non-English language country setting [27]. The two raters in our study read relevant literature about AGREE II Instrument and its applications and discussed individual items before actual scoring. They were consistent in their scores and their agreement was good or very good, as judged by kappa statistics.

The finding of low scores for Croatian guidelines in most of AGREE domains (“Rigour of Development”, “Stakeholder Involvement”, “Applicability” and “Editorial Independence) is similar to the finding for other national guidelines, as recently published for the general population of guidelines and specific otorhinolaryngology guidelines in China [3], [4]. Like in China, Croatian guidelines have mostly been developed as expert opinions, which generally followed the methodology and presentation of a literary review, rather than methodologically rigorous analysis of evidence. This is probably the reason why guidelines derived by Croatian professional scored zero for “Editorial Independence” domain, while the guideline from a European organization, adopted by Croatian professionals, scored better on this domain (38%). EFNS guideline scored higher also on “Rigor of Development” and “Applicability” domains, indicating better methodological approach of professional groups creating guidelines at an international level, although the actual scores for the EFNS guideline leave a lot of space for improvement in methodological quality. A recent review of methodological quality of 28 national, as well as pan-European guidelines for the management of chronic disease in Europe demonstrated lower scores for “Editorial Independence” “Applicability”, “Stakeholder Involvement” and “Rigour of Development” [8]. At both the European and national levels, there seems to be little coordination and methodological management of guideline development, leading to a non-transparent, decentralised and non-systematic approach to guidelines [7]. Such situation does not help local/national health care communities, who could benefit from adopting international clinical practice guidelines and adapting them to local specificities instead of spending resources in producing their own guidelines.

Professional societies in neurology and other clinical disciplines, both at national and international levels, should take seriously the finding from our and other studies on the methodological quality of clinical practice guidelines. At a national level, a good approach may be the translation of AGREE instruments and other relevant documentation [27], which should be a part of professional training. Professional associations could provide leadership for such training and for systematic collection of data on the development and quality of guidelines. Even more effective approach to build the capacity of the medical profession for high quality clinical guidelines and their implementation in practice, would be education in research methodology and evidence-based medicine at the level of medical schools [28], [29].
